# Modelling emergency response times for Out-of-Hospital Cardiac Arrest (OHCA) patients in rural areas of the North of England using routinely collected data

**DOI:** 10.1186/s12873-025-01170-7

**Published:** 2025-01-11

**Authors:** Megan Harries, Anastasia Ushakova

**Affiliations:** Lancaster University, Lancaster, UK

**Keywords:** Emergency services, Routinely collected data, Response times, Cardiac arrest

## Abstract

**Background:**

National response time targets for ambulance services are known to be more strongly maintained in urban areas compared to rural. That may mean that responses in rural areas could be less immediate which can in turn affect survival of those experiencing cardiac arrest. Thus, analysis of variation in response times using routinely collected data can be used to understand which rural areas have the highest need for emergency intervention. In this study we have focused, given the heterogeneity of demographic make up, on a specific area of the North of England. Some areas in North England have shown to have a large proportion of cardiac arrests occurring in a rural setting, specifically, in the anonymised study region this was almost half of the cases at 46.3%. Response times to these areas were found to be over 3.5 minutes slower than for urban areas making it worthy of further exploration.

**Methods:**

A retrospective observation analysis was conducted on routinely collected data from regional ambulance services for areas within the North of England from April 2016 to March 2021. Information was collected on service and geographic characteristics for 1915 incidents. A multivariable linear mixed effect regression model was used to understand the association between geographical, service factors and response times to cardiac arrest patients. To advance previous research which up to now only used visualisations to analyse ambulance response times, the study used a mixed effects model with a variety of predictors, capturing geographical variation alongside service characteristics.

**Results:**

From the cases analysed it was found that the mean response time to scene was 9.1 minutes, with a standard deviation of 6.4 minutes. After adjustment for geographic variation and incorporating robust standard errors into the model: distance to the nearest ambulance station (coefficient = 0.61, 95% confidence interval [CI]: 0.56-0.66), urgency of the call (Category 2, second most urgent, compared to the most urgent coefficient = 1.66, 95% CI: 1.13 - 2.18), location of the nearest ambulance station to the incident and the type of crew who attended the incident (Advanced Paramedic when compared to just Paramedic, coefficient = -0.70, 95% CI: -1.24 - -0.16) were all factors which affected response times to scene.

**Conclusion:**

For each extra km the incident was away from an ambulance station, the response time to scene increased by 37 seconds. The ambulance station which displayed the largest increase in response time, Station L was 170 seconds (95% CI: 79, 261) longer than Station N, which had a median performance across all stations, as measured by median survival rate to return of spontaneous circulation (ROSC). The rural geography of the North of England means that lots of cardiac arrest incidents occur a considerable distance away from the stations, emphasising the need to use alternative emergency services technologies within these rural areas to attend to patients sooner.

**Supplementary Information:**

The online version contains supplementary material available at 10.1186/s12873-025-01170-7.

## Background

Out-of-Hospital Cardiac Arrest (OHCA) cases are one of the most time-critical incidents ambulance services respond to. A response time is calculated as the time from the emergency call being answered to the time Emergency Medical Service (EMS) staff arrive at the scene of the incident. The mean target response time for ambulance crews in England for the most urgent, Category 1 (C1) calls, is 7 minutes, with a target of 90% of calls having a response time of less than 15 minutes [1]. The average ambulance response time for C1 calls throughout the UK was 6 minutes 54 seconds during 2021 [2]. An increased distance to hospitals was found to be associated with an increased risk of death in emergency situations [3], one of these being cardiac arrests.

Previous work has shown that it is of value for ambulance services and the wider National Health Services (NHS) to utilise routinely collected data to understand how services can be improved. It has been found that reducing ambulance response times to 5 minutes could almost double the survival rate for cardiac arrests not witnessed by ambulance crews [4]. One of the especially useful aspects which can be explored with such data are the effects of different demographic and geographical characteristics.

Previous research has shown that in some regions national response time targets are more strongly maintained in urban areas [1], potentially due to a higher density of hospitals and ambulance stations and better road networks. In Norway [5] and Taiwan [6] it has been found that urban areas are associated with higher short-term survival rates from OHCA, with shorter EMS response times also noted.

The focus of this paper is to investigate the predictors of responses time to cardiac arrests around the rural areas in the North of England, with its hilly regions and a road network that is difficult to traverse, leading to longer than average response times. The target mean response time for C1 calls is 7 minutes [3]. Nationally, the UK meets this target, however for the area studied, the average across recent years (April 2016 to March 2021) was significantly longer at 8 minutes 29 seconds. This study is the first to explore how geographical factors may explain this difference in response times using a comprehensive analyses of routinely collected data.

## Data

The data used within this research is a combination of routinely collected Ambulance datasets, merged with the Out-of-Hospital Cardiac Arrest Outcomes (OHCAO) registry, which is completed in partnership with all 14 ambulance trusts in the UK and retains information on all cardiac arrest incidents which occur out of hospital, are attended by an ambulance service organised response and cardiopulmonary resuscitation (CPR) is attempted [7]. Open-source datasets from the Office for National Statistics (ONS) have provided additional information such as the Index of Multiple Deprivation [8] (IMD) which measures relative deprivation for small areas in the UK, starting with the most deprived area (ranked at 1) to the least deprived and is characterised through aggregation of information on income, housing, employment, education and health, among others [9]. Rural/urban classifications [10] are also utilised. They are split into ten categories, with the incidents from our data falling into six of below:

- Urban city and town [in a sparse setting]
- Rural town and fringe [in a sparse setting]
- Rural village and dispersed [in a sparse setting]

The rural/urban classification is linked to the dataset through each Middle layer Super Output Area (MSOA), these are geographical regions which have an average population of 7,200 people. For the analysis, the categories are grouped on a higher level to either ’Rural’ or ’Urban’, with 46.3% and 43.7% of incidents respectively in each.

**Fig. 1:**
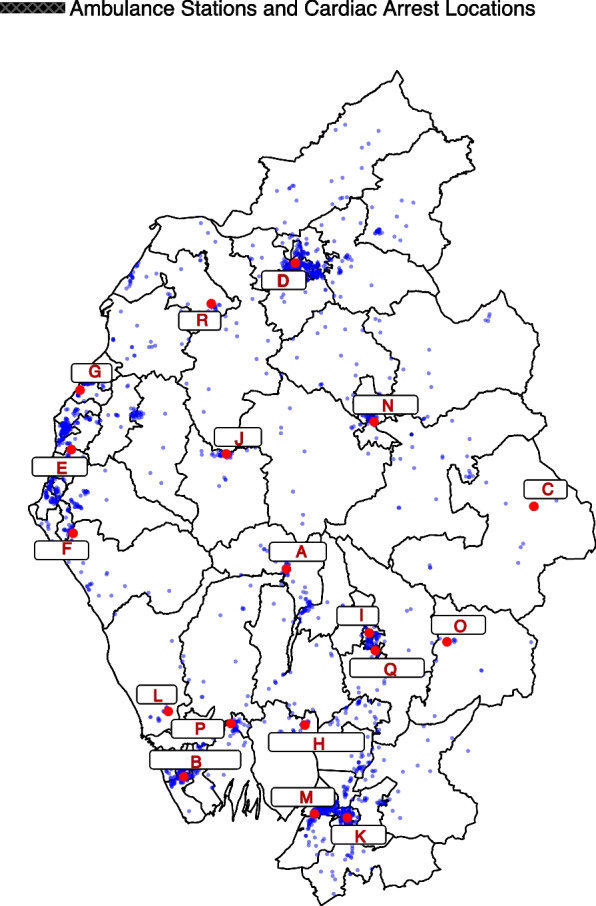
Locations of each ambulance station within the region were identified (shown in red) and incorporated into the dataset. The nearest ambulance station to each incident was identified using the Haversine formula, through using longitudes and latitudes to calculate the shortest distance between two points on a sphere. Cardiac arrest incidents are shown in blue

Overall, 1915 clinically classified cardiac arrest cases are analysed in the study area, spanning a five year period from 1st April 2016 to 31st March 2021. The data for each case has a unique identifiable call number assigned to it alongside variables recording various information on the location of the cardiac arrest, response time, IMD score of the incident location, type of crew attending the scene (e.g. advanced, senior or consultant paramedic, doctor or emergency medical technician (EMT), patient demographics, and survival. The dataset is grouped geographically and includes cases from 84 MSOAs or alternatively 50 postcode districts. Figure 1 displays the geographical spread of the incidents within each MSOA.

It is required for incidents to fulfill certain criteria for inclusion in the analysis. These criteria include: cases within the 50 study postcode districts, patients older than one year [11] and cases where ambulance service crew attended the scene and either identified the patient as having a cardiac arrest, or where a resuscitation attempt was made. Patients diagnosed as deceased on arrival or with a do not attempt cardiopulmonary resuscitation (DNACPR) order were not included in the OHCAO registry, alongside patients whose arrest was witnessed by a bystander, received bystander CPR and achieved return of spontaneous circulation (ROSC) before EMS arrived.

An overview of the dataset alongside descriptive statistics on relevant variables is provided in the Supplementary Tables. The data was largely full and complete. There was minor occurrence of missing-ness, namely in the data capturing category of the call. Nevertheless, this missing-ness was at less than 1% of total data, having no impact on data representativeness.

The response time to the scene of an incident recorded for C1 calls (84.1%) is the difference between the clock start time and the clock stop time. The clock starts with the earliest of: the call being coded; the first resource assigned or 30 seconds from call connect. The clock-stop time is recorded as the time when ambulance service crew arrive within a 200-metre geo-fence of the patient or if they confirm arrival at scene [12]. For C1 calls, the clock can also be stopped when an approved first responder deployed by the ambulance service, arrives within a 200-metre geo-fence of the patient, or confirms arrival at scene.

**Fig. 2:**
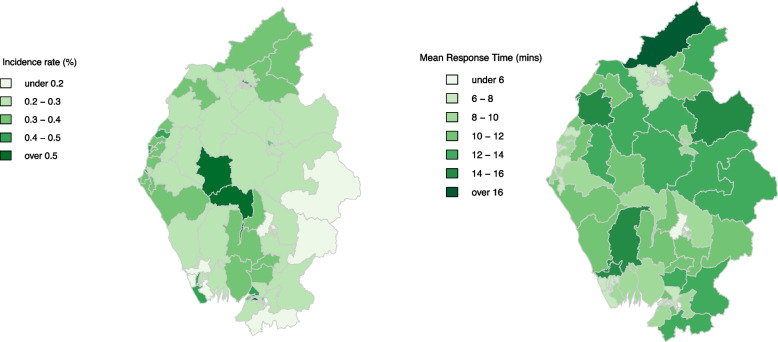
**a** OHCA incidence rate, the highest rates surround popular tourist areas which also contain an aging population. **b** Mean response times to cardiac arrest cases throughout the study period, with the longest response times seen on the northern border of the study area

The incidence rates of cardiac arrests during the study period within the population of each MSOA is shown in Fig. 2, the highest incidence rates are seen around popular tourist areas, attracting a large visitor population alongside containing aging resident populations. The mean response times across the MSOAs researched are shown in Fig. 2, with the longest mean response times observed on the northern border of the study area, with shorter response times consistently recorded in the most densely populated and urban areas.

The mean response time was 9.1 minutes (SD: 6.4 minutes) with 50.9% of arrests witnessed by a bystander, 65.2% receiving bystander CPR and defibrillation was administered by ambulance service staff in 36.7% of incidents. Cardiac arrests occurred at home in 78.4% of cases, with public automated external defibrillators (AEDs) available nearby for 21.8% of all cases within the study period. Many patients (42.2%) were not conveyed to a hospital due an unsuccessful resuscitation outcome. 46.3% of incidents occurred in rural areas with the mean distance to the nearest ambulance station at 4.4km (SD: 4.1km). The IMD scores for the areas were consistently spread, although skewed slightly towards the most deprived scores, with 34.6% of patients having a score of 1–3, 43.7% with 4–7 and 21.7% with 8–10 as seen in the Supplementary Tables.

## Methods

To understand the relationships between response time (seconds) and predictors, a mixed linear regression model was used. Ten dependent variables were used as covariates in the model: crew, IMD, category of call, location, urban/rural classification, MSOA population, hour, day of the week, nearest ambulance station and the distance to the nearest station. It is vital to note that the reference groups for the categorical variables were chosen such that the results of the model are easiest to interpret: the group containing the most cases - a Paramedic crew, C1 call and most cases occurring at home and in urban areas or the middle category in the variable - an IMD score of 5 and Station N having a median performance out of the ambulance stations when measured by survival. The type of crew dispatched to the scene can describe the experience of the responders whilst also providing information on the vehicle type used, which was likely to influence response times, as a senior or advanced paramedic may have used a rapid response vehicle, which is likely to have less trouble traversing rural roads. Advanced paramedics are expected to be closer to arrest cohorts, as they are usually based at urban stations or larger hub stations in rural areas. The variable for the nearest ambulance station was included to understand geographical effects and by proxy capture information around potential demand associated with each station. This however does not reflect the exact location of a resource at clock start time but provides an opportunity to capture clustered variation related to the stations individual characteristics. The inclusion of the call category was also vital in capturing the influences of the response urgency, and therefore the response time. Lower urgency calls, which then result in a cardiac arrest occurring, may have a slower response.

To appropriately account for spatial dependencies in response times we have used random effects for each MSOA, this accounts for similar roads encountered for each incident within a collective small region, alongside the regional variation in staff and vehicle levels across the ambulance service.

As a part of our investigation, we anticipated that interaction terms may be present - we extended the model through exploring interaction effects to identify if there is evidence to suggest conditional effects being present. The interactions studied are (Nearest Station * Nearest Station Distance), alongside the category of the call and the type of crew who attended the scene (Category * Crew).

To select the best model, the likelihood ratio test was used to compare the fit of the model with nested models through forward selection, including additional variables with significant *p*-values of less than 0.01. The model fit was evaluated using the R-squared value. To evaluate if there is a need to account for temporal dependency in the outcome variable, the stationarity of the data was also tested through the Augmented Dickey-Fuller test. The key assumptions for linear regression such as a linear relationship existing between the independent variables and the response, explanatory variables have no multi-collinearity, the residuals from the model are independent of each other and homoscedasticity - were checked as a part of our analyses.

## Results

The Augmented Dickey-Fuller test rejected non-stationarity in the data, therefore no time series elements were added to the model, with no seasonal effect identified. The likelihood ratio test identified that the best model for response times (*p*-value < 0.001 as shown in Table 1) included variables for distance to and the location of, the nearest station, the category of the call and the crew who attended the scene. The interaction terms between the nearest ambulance station and its distance, alongside the category of the incident and the crew who attended were also tested for inclusion in the model; the respective likelihood ratio tests did not provide evidence to suggest model improvement from such additions (*p*-values < 0.01), therefore these were not included in the final model. As shown by the confidence intervals in Table 2, the category of the call and the distance to the nearest station are both significant coefficients.

**Table 1:** P-values of the likelihood ratio test are shown compared to the previous best model

Variables	Log-likelihood	DF	*p*-value
(intercept only)	−6257	2	
1 + 1|MSOA	−6187	3	<0.001
Nearest Station Distance + 1|MSOA	−6112.2	4	<0.001
Nearest Station Distance + Category + 1|MSOA	−6050	7	<0.001
Nearest Station Distance + Category + Nearest Station + 1|MSOA	−6012	24	<0.001
**Nearest Station Distance + Category + Nearest Station + Crew + 1|MSOA**	**−6000**	**29**	**<0.001**
Nearest Station Distance + Category + Nearest Station + Crew + IMD + 1|MSOA	−5990	38	0.017
Nearest Station Distance + Category + Nearest Station + Crew + Location + 1|MSOA	−5999	30	0.203
Nearest Station Distance + Category + Nearest Station + Crew + Rural/Urban + 1|MSOA	−6000	30	0.564
Nearest Station Distance + Category + Nearest Station + Crew + Hour + 1|MSOA	−6003	30	0.021
Nearest Station Distance + Category + Nearest Station + Crew + Day of Week + 1|MSOA	−6002	30	0.059
Nearest Station Distance + Category + Nearest Station + Crew + MSOA Population + 1|MSOA	−6002	30	0.082

Model selection for linear regression mixed effects model

**Table 2:** Significant station and crew coefficients are emphasised

	Reference	Coefficient	Lower limit	Upper limit
**Intercept**		5.94	5.09	6.78
**Nearest Station Distance**		0.61	0.56	0.66
**Category**				
C2	C1	1.66	1.13	2.18
C3		3.63	2.26	5.01
Other		2.74	1.35	4.12
**Nearest Station**				
Station A	Station N	0.88	−0.32	2.08
Station B		−1.10	−2.08	−0.12
Station C		−0.70	−2.70	1.29
Station D		−0.90	−1.76	−0.04
Station E		−1.74	−2.72	−0.77
Station F		−0.62	−1.63	0.39
Station G		0.67	−0.34	1.68
Station H		1.02	−0.02	2.23
Station I		−0.59	−1.85	0.66
Station J		1.05	−0.28	2.37
Station K		−0.54	−1.44	0.36
Station L		2.84	1.32	4.35
Station M		−0.43	−1.39	0.54
Station O		−0.30	−2.20	1.60
Station P		−0.28	−1.66	1.10
Station Q		−0.21	−1.54	1.11
Station R		0.14	−1.07	1.35
**Crew**				
Advanced Paramedic	Paramedic	−0.70	−1.24	−0.16
Consultant Paramedic		0.49	−2.07	3.04
Doctor		0.71	−0.11	1.53
EMT		0.46	−3.93	4.86
Senior Paramedic		−0.33	−0.72	0.07

Robust linear regression results with 95% confidence interval of coefficients

The overall explanatory power of the model (R-squared = 0.37) suggested moderate ability of the set of predictors to explain variation in the outcome.

**Fig. 3:**
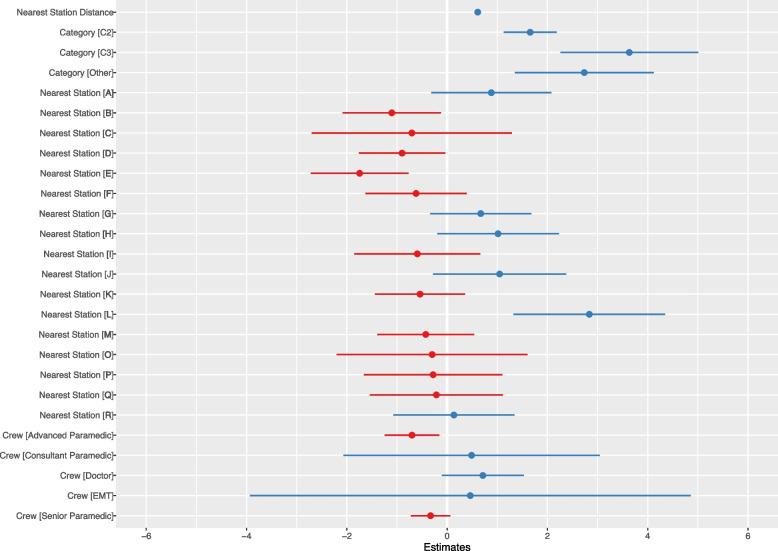
The linear mixed effects model with robust standard errors suggests variables which correlate to the response time as distance to the nearest station, category of the call, the crew who attended and nearest station location

Coefficient estimates and confidence intervals are provided for this linear mixed effects model with robust standard errors in Table 2 and visualised in Fig. 3. Robust to heteroscedasticity standard errors were included in the final model results.

Overall, we can see the key variables explaining the variation in response times are those related to geographical locations, with the distance to the nearest station predicted to increase the response time by 37 seconds, given by the coefficient of 0.61 $\times$ 60, (95% CI: 34 - 40) for each kilometre. The category of the call is associated to response times, as expected, an inverse relationship between the urgency of the call and the coefficient in the model seen.

The response time decreases significantly from the response time for Station N, for stations B, D and E, whilst Station L having the largest significant increase in response time at 2 minutes 50 seconds (95% CI: 1 minute 19 seconds, 4 minutes 21 seconds). Response times also decrease significantly when the first crew arriving is an advanced paramedic, in comparison to a paramedic (coefficient = −0.70, 95% CI: −1.24 - −0.16).

**Fig. 4:**
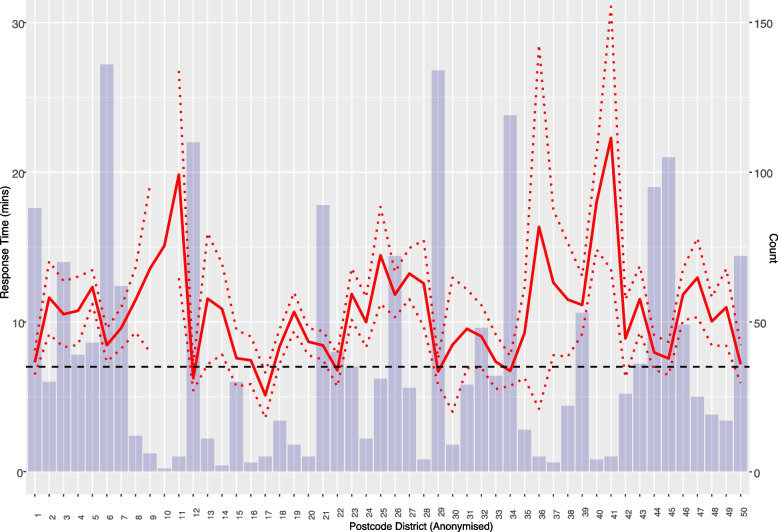
Mean response times with 95% confidence interval and incident counts for each postcode district. The red solid and dotted lines show the mean response time and the 95% confidence interval respectively. The black dashed line provides the baseline target response time (7 minutes). The blue bars show the count of the incidents within each postcode district

As shown in Fig. 4, which is useful for complementing regression results, the average response time and 95% confidence interval differ considerably by area, hence why a mixed effect model with a random intercept term to account for location was suitable for the data. Figure 4 shows that the mean response time for the majority of postcode areas within the study region (45, 90%) exceed the target response time of 7 minutes. Some notably long response times with means of over 14 minutes were seen for predominantly rural districts. Short response times, with a mean of less than 7 minutes, were seen for urban regions, with these districts having similarities in all containing or being close to an ambulance station. Postcode district is used in Fig. 4 in contrast to MSOAs used within the model, as the non-mapped geographic variation is easier to visualise at this granularity to highlight potential similarities across areas which are located nearby. MSOA captures smaller geographies in comparison to postcode areas [13], which tend to be more advantageous when capturing geographical boundaries and regional differences related to the socio-demographic composition of the population.

## Discussion

Using routinely collected data for a retrospective observational analysis of response times to OHCA in the North of England, we demonstrated an effect of the location of the OHCA and distance from the nearest ambulance station, the crew who attended the scene and the category of the call on response times through using a linear mixed effects model.

After rigorous step-wise model selection procedure, we have noted that some of the significant predictors of variation in response times in the model were spatial locations. Spatial locations were expected to influence response time significantly, as the ‘distance to the nearest station’ was the first variable identified in model selection, this also aligns with previous research [3]. The category of the call was also expected to be included in the model as this influences response time, due to C3 calls requiring a less urgent response than C2 calls, with C1 calls being prioritised. The type of crew dispatched to the scene also affected response times as it in turn may have affected the vehicle used, as a senior or advanced paramedic may have used a rapid response vehicle to travel to the incident, instead of an ambulance, which could have less trouble traversing the roads in rural areas.

The random intercept with the highest value was for an MSOA with long response times as seen in Fig. 2, whilst the lowest random intercept was for an MSOA which lies centrally between nearby ambulance stations, Stations E and F, whilst also located close to a hospital, so many ambulances are often nearby. The results from the model indicated that the station with the highest predicted response times was Station L, this station is likely to be connected to incidents with long response times as it is the nearest ambulance station to a large proportion of a National Park.

During model selection, IMD was not identified as significant for response time prediction as shown in Table 1, this may be because the nearest station and MSOA, which were already included in the model as random effects, capture the variation caused by unobserved geographical characteristics that would be also described by the deprivation score. Including both may be associated with potential multicolinearity and our analyses have shown that model with random effects for MSOA and station is indeed the best model.

One of the key factors to increase the chances of survival from cardiac arrest is the speed at which CPR commences and defibrillation begins. It has been shown that bystander CPR and the use of a defibrillator increase the survival rate from 8% to 32% [14]. Both CPR and defibrillation must begin as soon as possible, for each minute after cardiac arrest occurs the chances of defibrillation correcting the heart to a normal rhythm falls by between 7–10% [15]. Therefore, if defibrillation occurs in the first 5 minutes after cardiac arrest, during the electrical phase, there is roughly a 50% chance of correcting the heart’s rhythm, however after this point, the odds decrease considerably. One potential way to improve the chance of survival following a cardiac arrest is ensuring defibrillators are on scene within the first five minutes, which is often before the ambulance crew arrives. It is suggested that concentrating NHS resources to improve response times across the board is unlikely to be a cost-effective option [16]. To combat this, one of the suggested emerging technologies is the use of drones to deploy an AED to the scene [11].

This study is the first to utilise a large routinely collected dataset from ambulance services in the North of England to understand service provision in the area using highly granular geography. This is also the first paper to use linear mixed effects modelling to investigate relationships between geographical locations and response time to compare rural and urban areas. As such, it is especially useful for the ambulance trusts who are interested with understanding how deviation from target response times may affect performance and what, if any, potential interventions can be used to address longer response times in specific areas. The results found in this paper, although investigating cardiac arrest data, may be generalised to response times to other urgent (e.g. Category 1) incidents within this area of North England and potentially across the country when it comes to understanding of differences between response times in urban and rural areas.

This study is not without limitations. These are mainly driven by the nature of the datasets: the OHCAO dataset did not include potential cardiac arrest patients who were not resuscitated by EMS; this could lead to exclusion of a large proportion of patients who could have potentially survived if they received intervention sooner. Therefore using the response times of only those patients who received EMS resuscitation could bias the data towards shorter response times. The restrictions on the spatial data available also influences the results, as the origin of the ambulances prior to arrival on scene is not known, therefore factors such as distance and location of the nearest ambulance station could be improved upon. Additional spatial data such as the height location of the incident would be beneficial, to understand if arrests occurring in multi-story buildings impact response times. It is expected that the stations in rural areas may have a lower number of resources relative to urban areas and this could have an impact on response time. To address this, including information indicating allocated resources at each ambulance station to provide services would allow for more robust findings. In our analyses, category 2 and 3 incidents were included in the analysis for completeness of the research. However, these categories may need to be considered with caution as at the time of the call they were not identified as cardiac arrests cases and would usually be associated with longer response time as a result. Inclusion of these categories, while important, may skew the data towards longer response times. Finally, using MSOA as a random effect has captured some of the spatial differences related to population sizes and other unobserved characteristics of the areas we considered. This was also done to preserve anonymity of our data provider. However, one could also consider additional information such as population sizes across areas and number of ambulances per region as a covariate to explore further. While the model and model selection procedures we used were considered appropriate for this type of data, additional analyses may include predicting survival as an outcome in a logistic model with response times and other covariates considered in this paper.

## Conclusion

This paper provided a comprehensive analysis of routinely collected data to understand which factors are associated with variations in response times when differences in geographical areas are accounted for in the modelling. We have shown that rural areas indeed may have longer response times, so intervention for those areas may be necessary through novel and cost-effective technologies. It has been explored how drone networks could provide an opportunity to reduce AED travel times, [17], the practicality and use of this emerging technology is already being explored by practitioners in the UK.

A deeper evaluation into urban/rural discrepancies could be studied through a comparison of this geographical study area and more dense urban areas covered by the ambulance service. Further research into ambulance response times could also identify if a disparity exists between the North and the South of England as it has been identified in published clinical outcome data [18] that there is considerable variability in survival to hospital discharge following OHCA between ambulance services (4.7% - 16.7% in April 2016). Understanding which areas tend to predict lower survival rates and longer response times ensures that improvements can begin in the most critical areas and inequalities in access to care due to geographical variation can be addressed.

## Supplementary Information

Supplementary Material 1.

## Data Availability

Data was not generated for research but was analysed for research. Source data can be requested from the relevant Ambulance Service through contacting the corresponding author.

## Abbreviations

AED: Automated External Defibrillator
C1-3: Category 1-3
CI: Confidence Interval
CPR: Cardiopulmonary Resuscitation
DNACPR: Do Not Attempt Cardiopulmonary Resuscitation
EMS: Emergency Medical Service
EMT: Emergency Medical Technician
IMD: Index of Multiple Deprivation
MSOA: Middle Layer Super Output Area
NHS: National Health Service
OHCA: Out-of-Hospital Cardiac Arrest
OHCAO: Out-of-Hospital Cardiac Arrest Outcomes
ONS: Office for National Statistics
ROSC: Return of Spontaneous Circulation
SD: Standard Deviation
UK: United Kingdom
