# Supplementary material for: Modelling emergency response times for Out-of-Hospital Cardiac Arrest (OHCA) patients in rural areas of the North of England using routinely collected data
Source: BMC Emerg Med. 2025 Jan 11;25:8. doi: 10.1186/s12873-025-01170-7 (PMC11724540; doi:10.1186/s12873-025-01170-7)
Supplement: Supplementary file 1 — Supplementary Material 1. [file 12873_2025_1170_MOESM1_ESM.pdf]

A Supplementary

Supplementary Table 1: Service Characteristics of the Data

	**N = 1915**
Response Time - mean (SD)	
Seconds	545 (381)
Minutes	9.1 (6.4)
First on Scene	
EMS	1674 (87.4%)
Non EMS	241 (12.6%)
Arrest Witnessed	
EMS	268 (14.0%)
Bystander	974 (50.9%)
No	673 (35.1%)
CPR	
EMS	268 (14.0%)
Yes	1248 (65.2%)
No	399 (20.8%)
Ambulance Service Defibrillated	
Yes	703 (36.7%)
No	1212 (63.3%)

Supplementary Table 2: Geographical Characteristics of the Data

	**N = 1915**
Location	
Home	1501 (78.4%)
Not Home	414 (21.6%)
Area IMD Score	
1 (most deprived)	258 (13.5%)
2	170 (8.9%)
3	233 (12.2%)
4	217 (11.3%)
5	214 (11.2%)
6	182 (9.5%)
7	225 (11.7%)
8	180 (9.4%)
9	140 (7.3%)
10 (least deprived)	96 (5.0%)
Public AED Available	
Yes	418 (21.8%)
No	1497 (78.2%)
Rural/Urban	
Rural	887 (46.3%)
Urban	1028 (53.7%)
Distance to Nearest Ambulance Station (km) - mean (SD)	4.4 (4.1)
